# Effects of Dietary Red Raspberry Consumption on Pre-Diabetes and Type 2 Diabetes Mellitus Parameters

**DOI:** 10.3390/ijerph18179364

**Published:** 2021-09-04

**Authors:** Stefani A. Derrick, Aleksandra S. Kristo, Scott K. Reaves, Angelos K. Sikalidis

**Affiliations:** Department of Food Science and Nutrition, California Polytechnic State University, 1 Grand Avenue, San Luis Obispo, CA 93407, USA; sderrick@calpoly.edu (S.A.D.); akristo@calpoly.edu (A.S.K.); sreaves@calpoly.edu (S.K.R.)

**Keywords:** red raspberries, western diet, type 2 diabetes mellitus, pre-diabetes, metabolic syndrome

## Abstract

Type 2 diabetes mellitus (T2DM) is a chronic metabolic condition characterized by glucose clearance abnormalities and insufficient insulin response. Left uncontrolled, T2DM can result in serious complications and death. With no cure available currently and the prevalence of major risk factors such as pre-diabetes and the metabolic syndrome continuously increasing, there is an urgent need for effective treatments with limited or no side effects. Red raspberries (RR) contain various phytonutrients with potential for modulating insulin function, glucose, and lipid metabolism. The objective of this literature review was to investigate the potential metabolic benefits of dietary RR in individuals with T2DM and pre-diabetes. A search of major scientific databases was employed to identify peer-reviewed, in vivo, or human studies that utilized whole RR or its functional constituents as treatment. The studies examined provide evidence that RR may offer clinically beneficial effects for the prevention and management of chronic diseases through improvements in glucose handling and insulin sensitivity, adiposity, lipid profiles, ectopic lipid accumulation, inflammation, oxidative stress, and cardiac health. More human trials and in vivo studies are needed to confirm the benefits of dietary RR in T2DM and pre-diabetes and to explore the dose-dependent relationships, optimal duration, and treatment modality.

## 1. Introduction

Type 2 diabetes mellitus (T2DM), is a chronic metabolic pathology, characterized by abnormalities in glucose clearance with chronic hyperglycemia and insufficient response to insulin, i.e., insulin resistance [1,2]. Adults and youth worldwide are affected by T2DM with clinical manifestations and disease progression varying substantially across individuals [1]. The diminished capacity for plasma glucose clearance induces the pancreas to compensate by secreting more insulin, resulting in hyperinsulinemia and an overburdening of the pancreas that can eventually lead to a decrease in β-cell functioning [2]. Chronic, uncontrolled hyperglycemia can lead to damage of tissues and organs, through excessive glycation phenomena, resulting in microvascular and macrovascular perturbations, in turn affecting the nervous system, eye, kidney, and heart. Serious complications due to long-term, uncontrolled diabetes include kidney disease, cardiovascular disease (CVD), amputation, blindness, and even death [3], leading to an increased need for medical care and loss of productivity in the long term [2].

Both modifiable and non-modifiable factors that place individuals at high risk for developing T2DM have been identified, most notably pre-diabetes, a condition characterized by abnormally elevated blood glucose [4], and the metabolic syndrome (MetSy), a combination of metabolic abnormalities that increase the likelihood of developing diabetes and cardiovascular disease (CVD) [5]. While a cure for T2DM remains elusive, current treatments include pharmaceutical and insulin therapies along with diet and lifestyle modifications aiming at optimal management of the disease [6].

Non-modifiable risk factors for T2DM include but are not limited to: age (with risk increasing after 40 y and greatly increasing after 60 y), sex (males appear more susceptible), genetics/family history of diabetes, ethnicity/race (e.g., African American, Hispanic, Native American, Asian American, and Pacific Islander), and in case of women, diagnosis of gestational diabetes [1]. Modifiable risk factors include: conditions associated with the MetSy, severe or morbid obesity (BMI ≥ 40), non-alcoholic fatty liver disease (NAFLD), other conditions related to insulin resistance, poor diet, and lack of physical activity. It is important to note that certain prescription medications, such as thiazide diuretics, atypical antipsychotics, glucocorticoids, and statin-type drugs, prescribed for high cholesterol and dyslipidemia, may also increase blood glucose levels placing the patient at risk for T2DM [1,7,8].

Globally, the prevalence of diabetes has grown from 108 million people in 1980 to 422 million people in 2014 with a rapid increase in countries experiencing epidemiological transition, greater compared to developed countries [9]. In 2018, 34.2 million adults and children in the United States (US) were living with diabetes and an estimated 7.3 million adults remained undiagnosed (21.4% of diabetic adults), with 90–95% of cases classified as T2DM [10]. With the national and global prevalence of diabetes growing rapidly, there is an urgent need to address this crucial public health issue.

The negative economic impacts of diabetes result not only from direct costs, such as increased healthcare utilization and hospitalization, but from indirect costs such as diabetes-induced disability, premature death, and absenteeism from education and work [11]. In 2019, 9.3% of the world’s adult population was living with diabetes, an estimated 463 million adults aged 20–79 y, while an additional 7.5% of the population, 374 million adults in the same age range, were living with pre-diabetes [2]. This translates to a global economic burden of USD 760 billion, an amount projected to rise to USD 825 billion by 2030. Investigating the efficacy of dietary interventions that could be utilized in lifestyle modifications for diabetics, such as including red raspberries (RR) in a balanced diet, may reveal potential benefits for public health as well as US agriculture [10].

Red raspberries (*Rubus idaeus* L.) constitute a low-glycemic index fruit containing a small amount of carbohydrates and relatively high amount of dietary fiber per weight unit [12]. Their phytonutrients and bioactive compounds may reduce the risk for chronic diseases and improve their management when consumed as part of a well-balanced diet. The major bioactive polyphenols found in RR are anthocyanins, which produce the brilliant red coloring of the berry, and ellagitannins/ellagic acid, found only in a few select foods [13]. The possible health-promoting benefits of RR consumption include improved insulin response, glucose, and lipid metabolism, as well as antioxidant and anti-inflammatory properties [12].

Two countries that may particularly benefit from foods that mediate the risks for and assist with management of T2DM are China and India. In 2019, China and India were estimated to have ≥20 million adults aged 20–79 y living with diabetes [2]. Along with the US, these countries have the highest rates of diabetes in the world, with numbers projected to grow substantially in the coming years. Due to potential health-promoting benefits of RR for T2DM and pre-diabetes, their production represents an immense opportunity for agriculture. Interestingly, in 2018, the US was the fifth largest producer of RR worldwide [14,15] which suggests significant potential for the US economy, including expanded export potential of this crop and an increase in its value, in promotion of RR as having evidence-based therapeutic uses in chronic diseases [13].

The objective of this literature review was to investigate the metabolic effects of dietary red raspberries in individuals with T2DM and pre-diabetes. However, due to the limited amount of human work, in vivo animal work was also considered. The review herein discusses the characteristics of red raspberries, including an overview of their bioactive compounds and proposed health benefits, as well as peer-reviewed research studies investigating the use of red raspberries as treatment for metabolic dysfunction. The institutionally developed “OneSearch” search engine, provided by California Polytechnic University’s Robert E. Kennedy Library, was utilized to search for peer-reviewed, in vivo, and human studies using the search terms: “red raspberries” or “raspberries” combined with “type 2 diabetes mellitus”, “diabetes”, “pre-diabetes”, or “metabolic syndrome”. The OneSearch search engine includes, but is not limited to, major scientific databases such as PubMed, Web of Science, and Scopus. Articles were excluded if they did not include animal models or human participants with T2DM, pre-diabetes, or metabolic syndrome, and if raspberries, or an extract of the berry, were not the primary treatment. The search also used the following inclusion criteria: manuscript written in English and published during or after 2010. While there is a significant body of literature available documenting the health effects of a variety of berries, the evidence is somewhat limited regarding red raspberries, hence the present work aimed as a scope to indicate in brief what the current evidence suggests in terms of the relationship between red raspberry consumption in natural or powdered/extract form and effects on metabolic syndrome, pre-diabetes and T2DM, while potentially revealing areas where further research is needed. The source search was performed during the period of: December 2020–April 2021. 

## 2. Red Raspberries: An Overview

The first records of the red raspberry (RR) date back to Troy in the first century A.D., with domestication and cultivation throughout Europe by the Romans in the fourth century, and utilization for art and medicinal purposes in medieval Europe. Today, these berries are cultivated worldwide by the metric ton with most of the production in the US from California, Washington, and Oregon [13]. While there are several varieties of raspberries, red raspberries constitute the most consumed variety and can be distinguished from other types of berries primarily by color and external texture [13].

Red raspberries can be consumed in raw or processed forms, such as frozen, dried, juiced, or powdered, as well as an extract of its bioactive constituents [12]. Main varieties of red raspberries include the following: Boyne, Heritage, Latham, Prelude, Bababerry, Candy, September, and Amity. Considered a low glycemic index food, RR contributes a minimal amount of carbohydrates and calories to the diet while providing an ample amount of dietary fiber and micronutrients, such as folic acid and vitamins C and K, magnesium, potassium, calcium, and iron. According to the United States Department of Agriculture (USDA) FoodData Central database, 1 cup (140 g) of frozen RR provides 70 kcal of energy and 17 g of total carbohydrates with 8.96 g of dietary fiber (6.4/100 g), thus constituting one of the highest sources of dietary fiber among whole foods [13,16].

### 2.1. Bioactive Compounds

Polyphenols with proposed biological activity contained in RR include anthocyanins, such as cyanidin and delphinidin, flavanols, flavonols, and phenolic acids, such as ellagitannins/ellagic acid [17]. Anthocyanins are plant pigments that give berries their red, blue, and purple coloring, with approximately 47 mg of anthocyanins per 100 g of RR [12,18]. Anthocyanins are of particular interest for mitigating chronic disease risk; however, their content appears to be greatly reduced during berry processing. For instance, anthocyanins are particularly sensitive to duration and temperature of storage and frozen berries tend to have an average of 42% less anthocyanins than their raw counterparts [12]. Another major polyphenol class found in RR are ellagitannins found in certain berries and nuts, muscadine grapes, and pomegranates. Like anthocyanins, ellagitannin content varies based on the storage and processing methods used. Ellagitannin bioavailability may be increased after conversion to urolithins by colonic bacteria, absorption by colonic enterocytes, and glucuronidation in the liver, a process with great inter-individual variability [19]. Although the bioavailability of both anthocyanins and ellagitannins is low and depends on several factors, such as the food matrix, dose and time of intake, interactions with gut microbiota as well as other polyphenols during digestion and absorption, there is increasing evidence that their biological activity may provide significant health benefits [13,20,21]. In addition to the bioactive compounds of RR there are post-ingestion metabolites such as short chain fatty acids that are shown to confer potential health benefits [22,23,24,25].

Dietary fibers are another important bioactive component of RR in that they promote growth of healthy gut microbiota [26], the composition of which has been shown to vary in individuals who are obese and/or have T2DM [21]. Dietary fibers including poly- and oligosaccharides remain undigested until reaching the large intestine where microbiota-derived metabolites such as short-chain fatty acids (SCFAs) are produced. Such metabolites can be utilized by the host for energy and decrease appetite through increased satiety and reduced production of the hunger hormone ghrelin, as well as other potential mechanisms under investigation [21,27].

### 2.2. Proposed Health Benefits

There is evidence that the benefits of dietary berries can be feasibly attained through daily consumption of 40–250 g of fresh, frozen, or dried berries, or their extracts [12]. Numerous health benefits have been documented from consuming RR and its bioactive constituents including improved glucose, insulin, and lipid metabolism, as well as antioxidant, anti-inflammatory and signaling properties which have the potential to reduce the risk for and modulate chronic metabolic diseases [12,23,24].

The proposed mechanism for the modulation of postprandial glycemia following consumption of dietary berries or their extracts is the inhibition of the enzymes α-amylase and α-glucosidase to prevent glucose absorption in the intestines [13]. RR extracts may be more effective at inhibiting α-amylase than other berry extracts, while anthocyanins, may enhance insulin secretion from pancreatic β-cells, thus improving insulin sensitivity [13].

Dietary berries may modulate lipid metabolism by increasing high-density lipoprotein cholesterol (HDL-c) through amplified synthesis of apolipoprotein-A1 in liver cells, decreasing triglycerides through down-regulation of transcription of genes involved in fatty acid synthesis, and through decreasing oxidative stress and inflammation in cells and tissues [25]. According to a meta-analysis of 32 randomized controlled trials, the attenuation of low-density lipoprotein cholesterol (LDL-c) was greater in participants with signs of dyslipidemia, with results being specific to anthocyanins purified from berry sources versus other food sources [26].

Most of the early research on RR focused on their antioxidant properties since oxidative stress and inflammation constitute important features of various chronic diseases, including T2DM and CVD [13]. An imbalance of reactive oxygen species (ROS) to antioxidants can lead to oxidative stress and damage to cells and tissues. Lipid peroxidation causes oxidized LDL-c accumulation, which can lead to plaque formation in arteries and stimulation of pro-inflammatory genes. This activation of the inflammatory response can lead to the inflammation observed in most chronic diseases [27]. Anthocyanins have been shown to act as antioxidants when ingested, causing increased synthesis of endogenous antioxidant enzymes such as glutathione peroxidase (GPx), catalase (CAT), and superoxide dismutase (SOD). However, anthocyanins like other antioxidant compounds may produce a pro-oxidant effect in very high doses. Those doses cannot practically be obtained via the diet, so the dietary intake of anthocyanins poses no concern. Such concerns might become more of an issue if high levels of supplementation are pursued. Anthocyanins have also been shown to reduce inflammatory cytokine gene expression in white adipose tissue (WAT) [28]. Ellagic acid has been shown to both reduce ROS production and inhibit the proliferation of oxidized LDL-c. Therefore, dietary berries and their bioactive compounds play a role in protecting cells and tissues during oxidative stress and assist in reducing inflammation [13,29].

## 3. Research Studies Investigating Dietary Red Raspberries as Intervention for Metabolic Conditions

### 3.1. Effects of RR Supplementation on Glucose Handling and Insulin Signaling

Altered glucose and insulin homeostasis and a decrease in insulin sensitivity are key indicators of metabolic dysfunction that can lead to T2DM [1]. In three independent randomized controlled trials (RCTs) whole red raspberry (RR) supplementation were utilized in adult participants with metabolic dysfunction (Table 1). Of those studies, Schell et al. (2019) and Xiao et al. (2019) assessed glucose and insulin homeostasis [30,31]. Both studies reported that 250 g of frozen RR consumed with a high-fat challenge breakfast meal elicited a decrease in plasma glucose levels and area under the curve (AUC) post-prandially in individuals with either T2DM or pre-diabetes and overweight or obesity (PreDM-O). However, no significant long-term benefit to blood glucose levels were found in participants with T2DM after consuming 250 g RR as a daily snack for 4 weeks [30]. Interestingly, doses of both 125 g and 250 g frozen RR prevented the plasma glucose dip below baseline at 2 h post-meal in the control group of metabolically healthy individuals, suggesting additional benefits of RR consumption for those without metabolic dysfunction [31]. In individuals with PreDM-O both doses of RR were effective in reducing plasma insulin AUC post-prandially, related to a time-shift of the insulin peak from 30 min in control to 1 h with either RR dose [30], while in T2DM patients no significant changes in serum insulin levels post-prandially were noted [31]. Thus, the bioactive components of RR may have a more pronounced effect on individuals with pre-diabetes compared to individuals with diabetes and insulin resistance (IR).

Seven animal studies were identified that utilized whole RR as a treatment for the consequences of a high-fat diet (HFD) and/or obesity, T2DM, or MetSy (Table 2). Four of them found that supplementation with whole RR, restored glucose homeostasis and improved insulin signaling and sensitivity altered by a high-fat diet (HFD) in various metabolically active tissues, including skeletal muscle, brown adipose tissue (BAT), and inguinal white adipose tissue (IngWAT) [32,33,34,35]. Insulin resistance promoted by a HFD was improved with RR supplementation, albeit only partial restoration of insulin signaling in IngWAT was seen [36]. Raspberry juice concentrate (RJC) was effective in lowering insulin levels raised due to a HFD, but not in lowering elevated fasting glucose levels [37]. Wild type (WT) mice fed a standard chow diet benefited from RR supplementation through induced insulin signaling [35]. However, in another study RR did not lower fasting blood glucose in Db/Db mice fed a standard chow diet and lead to increased plasma resistin levels, which could lead to the promotion of IR in mice genetically susceptible to obesity and T2DM [38]. Since limited information on the role of resistin is available through measurements of resistin levels, further investigation is warranted. The same group demonstrated that RR decreases heart biomarkers of cardiac remodeling with associated with oxidative and inflammatory stress in obese diabetic mice [39]. Taken together, these results illustrate the potential for whole RR supplementation for improving altered glucose handling and insulin resistance secondary to a HFD and/or metabolic dysfunction.

Five animal studies identified utilized RR functional components or extracts as treatment for the consequences of a HFD, high-fat and high-fructose diet (HFFD), or high-fat and high-sucrose diet (HFSD; Table 3). In these studies, a HFFD, HFD, and HFSD produced dysregulation of glucose and insulin metabolism [40,41,42]. Attia et al. observed significant decreases in fasting glucose and insulin levels with raspberry ketone (RK; 55 mg/kg) supplementation, and with supplementation of a combination of RK and *Garcinia Cambogia* (GC; 600 mg/kg) which caused a decrease in fasting glucose even further to levels comparable to that seen in the low-fat diet (LFD) control group. Insulin resistance (IR) was promoted by the HFFD and improved by both the RK and RKGC combination treatments [40]. Additionally, fasting glucose and insulin levels, as well as IR were significantly decreased, with a significant increase in quantitative insulin sensitivity check index (QUICKI), by both a low- and high-dose RK supplement (250 mg/kg and 500 mg/kg, respectively), with even greater decreases observed in the higher dosage [41]. Increased fasting insulin levels, glucose, and insulin AUCs and IR, all caused by a HFSD, were mitigated by supplementation with raspberry seed flour (RSF) [42]. Raspberry-derived polyphenols were shown to inhibit α-amylase in vitro thus with potential implications for T2DM [43], while the inhibitory effect of raspberry ketone on α-glucosidase was demonstrated through in silico docking analysis [44]. Thus, various functional components of RR appear to have beneficial effects on glucose handling and insulin sensitivity in several experimental settings.

### 3.2. Effects of RR Supplementation on Adiposity

Increases in body weight, excessive lipid accumulation in white adipose tissue (WAT), and ectopic lipid deposition in peripheral organs have been shown to contribute to metabolic dysfunction [1]. Animal studies utilizing whole RR as a treatment for the consequences of a HFD and/or obesity, T2DM, or MetSy demonstrated a shift in overall fuel utilization to lipid metabolism that has potential benefits for reducing adiposity (Table 2). While Zhao et al. (2018) and Xing et al. (2018) found increased mitochondrial biogenesis in skeletal muscle and IngWAT, respectively, with RR treatment, Zhu et al. (2018) reported a decrease in liver mitochondrial biogenesis without any changes in respiratory exchange ratio (RER) compared to HFD alone, suggesting that lipids remained the preferred fuel source [33,35,36]. Supplementation of a HFD with RR was shown to prevent excess weight gain, decreased fat accumulation in skeletal muscle, and decreased WAT mass in different rodent models [33,34,36,37]. Conversely, Zhu et al. showed only a decreasing trend in excess body weight in WT mice fed a HFD supplemented with RR and Noratto et al. (2017) found no change in body weight, BMI, body fat mass, or organ weight in obesity-prone mice fed a standard diet supplemented with RR [35,38]. Additionally, Zou et al., utilizing RR, demonstrated increased BAT mass in AMPKα1 knockout C57BL/6 mice and, along with Xing et al., showed browning of WAT with increased thermogenic gene expression and protein content, along with decreased hypertrophy and increased hyperplasia of adipocytes in WAT in C57BL/6 mice [34,36]. Two of the animal studies utilizing whole RR as a treatment for consequences of a HFD also measured indicators of basal metabolic rate (BMR) in WT mice. While Zou et al. observed that RR supplementation increased VO_2_, VCO_2_ (*p* < 0.1), heat production, and O_2_ consumption in IngWAT with a decrease in RER, Zhu et al. found increased VO_2_ and VCO_2_ with no change in heat production and static, low RER [34,35]. Both studies indicate that RR supplementation led to a slight increase in BMR, while lipids remained the preferred fuel source. Combined, the findings from in vivo studies investigating the effects of whole RR supplementation suggest that such intervention produces a shift to utilizing excess lipids as fuel, hence resulting in reduced body weight and fat mass with increased thermogenesis. 

Animal studies utilizing RR functional components or extracts as treatment for the consequences of a HFD, HFFD, or HFSD demonstrated beneficial effects on adiposity (Table 3). A HFD or HFFD promoted obesity and increased WAT weight in Wistar and Wister albino rats, as well as C57BL/6J mice [40,41,45]. Attia et al. provided evidence that raspberry ketone (RK) supplementation (55 mg/kg) partially ameliorated obesity, visceral adipose tissue (VAT) congestion and swelling, and adipocyte hyperplasia, with capacity to further decrease adipocyte area compared to a LFD [40]. The combination of RK with GC (600 mg/kg) was also shown to slightly ameliorate obesity and adipocyte hyperplasia, and to fully restore normal VAT structure, thus eliminating congestion and swelling. Tu et al. showed that red raspberry extract (RRE) supplementation partially reduced body weight gain and WAT weight gain induced by a HFD and down-regulated hepatic gene expression of *Acsl3*, suggesting a mechanism for decreased fat accumulation in the body and liver [45]. However, other findings suggested a dose-dependent relationship as only medium-dose RRE (200 mg/kg) and high-dose RRE (300 mg/kg) decreased WAT fat indices and cell size, while only the high-dose RRE decreased overall body weight [45]. Mehanna et al. found that both low-dose (250 mg/kg) and high-dose (500 mg/kg) RK (both doses higher than those utilized in the Attia et al. study), reduced body weight gain and adipose tissue weight increased by a HFD, while adipocyte cell size was partially reduced by low-dose RK and fully reduced to a level comparable to that of the standard chow diet by high-dose RK in Wister albino rats [41]. Conversely, Kang et al. (2016) reported no mitigation of body, liver, or WAT weight increased by a HFSD from raspberry seed flour (RSF) supplementation [42]. All experimental doses of RK (55 mg/kg, 250 mg/kg, and 500 mg/kg) used in two studies were found to be effective at lowering serum leptin levels and restoring them to physiological levels [40,41]. Additionally, serum STAT3 was decreased partially by RK and even further by the combination of RKGC [40], while a low dose RK lead to improved levels of adipokines (apelin, visfatin, and adiponectin) and WAT gene expression (leptin, apelin, apelin receptor, visfatin, adiponectin, and aquaporin-7) [41]. Thus, the functional components of RR appear to be effective at decreasing adiposity caused by high-energy diets and improving adipose tissue metabolism.

Of the RCTs with human participants (Table 1), only one study measured serum adipokines in adults with symptoms of MetSy [46]. A daily treatment of 300 g of combined whole strawberries, raspberries, and cloudberries (SRC) increased serum leptin levels over time, with no change in resistin levels compared to a control group with restricted berry consumption, suggesting that combined berry intake may suppress appetite and regulate excess energy consumption [37]. Weight loss due to reduced energy consumption has been shown to ameliorate MetSy and therefore has the potential to prevent progression to conditions such as T2DM and CVD [5]. Additionally, consumption of SRC was positively correlated with production of urolithins and alteration of gut microflora, responses not seen in the control group with restricted berry intake [46]. Urolithins are the metabolites of ellagitannin digestion in the colon shown to extend biological activity [13]. Thus, intake of polyphenol-rich berries increases the production of the bioactive components and may contribute to desirable alterations in gut microflora that enhance bioavailability. 

### 3.3. Effects of RR Supplementation on Lipid Profiles

Dyslipidemia is a characteristic of the MetSy and an associated risk factor for developing T2DM [5]. Several trials conducted with human participants measured blood lipid profiles after RR supplementation both as addition to a high-fat challenge meal and as a daily long-term treatment (Table 1). Only a decreasing trend was observed in serum triglycerides (TG) in individuals with T2DM post-prandially, but no change in serum lipoproteins after a meal, nor serum TG or lipoproteins with long-term supplementation of 250 g RR were documented [30]. Other studies reported no change in the postprandial lipid profiles of individuals with PreDM-O with 125 g of RR, or in serum lipids in individuals with MetSy from SRC supplementation [31,46]. Interestingly, 250 g of RR consumed with a high-fat challenge meal significantly increased plasma TG post-meal compared to 125 g RR and the control in participants with PreDM-O, results not seen in the metabolically healthy reference group, suggesting possible dose-dependent adverse effects in those with altered lipid metabolism [31]. Further research is needed to investigate the safety of supplementing 250 g RR to a high-fat diet in individuals who are pre-diabetic and/or those with dysregulated lipid metabolism at risk for CVD. 

Four of the animal studies that utilized whole RR as a treatment for the consequences of a HFD and/or obesity, T2DM, or MetSy assessed blood lipid profiles (Table 2). Kirakosyan et al. (2018) and Zou et al. (2018) independently demonstrated that RR supplementation was effective at decreasing serum lipids to levels significantly lower than that of the HFD alone, while Zhu et al. observed only a decreasing trend in serum lipids [32,34,35]. Additionally, RR increased gene expression of adiponectin receptor 1 (*Adipor1*) and decreased gene expression of lipoprotein lipase (*Lpl*) in cardiac muscle, suggesting improved lipid metabolism and decreased capacity for lipids to enter cardiac muscle [32]. Contrastingly, Db/Db mice, prone to obesity and T2DM, fed a standard chow diet supplemented with RR, exhibited increased plasma total cholesterol (TC) and LDL-c levels, compared to mice fed a standard chow diet alone, with a non-significant increase in HDL-c and no change in the atherogenicity index [38]. These findings indicate, similarly to the findings of the RCT by Xiao et al. (2019) [31] mentioned previously, a possible dose-dependent adverse effect of RR on lipid metabolism in individuals with a propensity towards obesity and diabetes. RR supplementation shows potential to improve lipid profiles altered by a HFD regime; however, further research is needed to clarify the safety parameters of such supplementation in individuals with altered lipid metabolism.

Four of the animal studies utilizing RR functional components or extracts as a treatment for the consequences of a HFD, HFFD, or HFSD demonstrated beneficial effects on blood lipid profiles (Table 3). The 55 mg/kg RK was beneficial at lowering serum levels of TG, TC, LDL-c, and free fatty acids (FFAs) and raising serum HDL-c altered by a HFFD, while the combination of RKGC improved levels of all serum lipids comparable to that in WT rats fed an LFD [40]. Similarly, both low- and high-dose RK (250 mg/kg and 500 mg/kg, respectively) were shown to reduce serum TG levels increased by a HFD and restore serum TC, LDL-c, and HDL-c to a level comparable to that in WT rats fed a standard chow diet [41]. Additionally, medium- and high-dose RRE (200 mg/kg and 300 mg/kg, respectively) restored levels of serum TG and TC increased by a HFD to levels comparable to that of WT mice fed a standard chow diet [45]. A HFSD diet increased plasma TC and LDL-c and lowered plasma HDL-c compared to a HFD in WT mice, while RSF supplemented to the HFSD led to significant improvement of the TC, LDL-c, and HDL-c levels [42]. Taken together, these findings demonstrate that several functional components of RR can improve lipid profiles altered by various types of “Western-style” high-energy (as per caloric density) diets.

### 3.4. Effects of RR Supplementation on Hepatic Steatosis

Ectopic lipid accumulation in the liver leads to a dysregulation of liver metabolism and constitutes a risk factor for altered glucose handling and development of T2DM [7]. Of the identified animal studies utilizing whole RR as a treatment for the consequences of a HFD, three studies assessed markers of hepatic steatosis (Table 2). A HFD diet induced ectopic lipid accumulation in the liver of WT mice [35,37], whereas RR produced a significant decrease in hepatic lipid droplets but not total TG in liver tissue, while RPC and RJC decreased liver lipid levels, although not to levels expressed by mice fed a LFD [35,37]. RPC and RJC significantly increased hepatic gene expression of TG catabolism and RJC alone showed increased expression of genes protective against oxidative stress in the liver [37]. Interestingly, liver lipid accumulation in mice prone to obesity and T2DM fed a standard chow diet remained unaltered after RR supplementation [38]. Therefore, RR supplementation appears to be most effective at mitigating ectopic lipid accumulation in the liver when caused by excess energy intake in a HFD. 

Animal studies utilizing RR functional components or extracts as a treatment for the consequences of a HFD or HFSD provided additional evidence of protection against excessive hepatic lipid accumulation (Table 3). The high dose of RRE (300 mg/kg) was determined as the most effective at decreasing hepatic TG, TC, and bile acid content, while lower doses were effective at lowering TG levels only [45]. In another study both low- and high-dose RK (250 mg/kg and 500 mg/kg, respectively) were effective at reducing liver weight index to levels comparable to a standard chow diet, while hepatic steatosis was only slightly ameliorated by low-dose RK [41]. Kang et al. (2016) demonstrated that a HFSD raised hepatic TG to significantly higher levels than a HFD alone; however, only a decreasing trend in hepatic TG was produced by RSF supplementation [42]. Positive improvements in hepatic gene expression related to endogenous cholesterol synthesis and metabolism, lipolysis, fatty acid (FA) synthesis, regulation of lipid metabolism, and gluconeogenesis were observed [42,45]. Serum markers of liver damage, alanine aminotransferase (ALT) and aspartate aminotransferase (AST), were also measured in the Tu et al. and Mehanna et al. studies. While Mehanna et al. observed decreases in serum ALT and AST raised by a HFD through high-dose RK supplementation and even greater decreases from low-dose RK, Tu et al. found that the HFD raised only AST levels which were non-significantly attenuated by medium- and high-dose RRE (200 mg/kg and 300 mg/kg, respectively) [41,45]. In all cases, RR functional component or extract supplementation was demonstrated to have protective effects against hepatic steatosis without contributing to liver damage. Moreover, a whole red raspberry enriched diet (8% *w/w* supplementation) for 8 weeks was hepatoprotective in the obese Zucker fa/fa rat (OZR), leading to reduced plasma cholesterol and HDL-c and increased plasma TG, while reducing hepatic TG accumulation [47]. Finally, raspberry ketone was shown to protect rats fed high-fat diets against nonalcoholic steatohepatitis [48].

### 3.5. Effects of RR Supplementation on Inflammation

Systemic inflammation and up-regulation of pro-inflammatory cytokines in organ tissues are characteristic signs of metabolic dysfunction [8]. Two RCTs assessed systemic inflammation in human participants with both acute and long-term RR supplementation (Table 1). Xiao et al. (2019) observed no beneficial postprandial effects of RR supplementation on systemic inflammation in PreDM-O participants; however, Schell et al. (2019) found that 250 g RR significantly decreased serum levels of interleukin 6 (IL-6) and tumor necrosis factor alpha (TNF-α) in individuals with T2DM both post-meal and long-term [30,31]. These findings imply that RR supplementation may be a more effective treatment for inflammation in individuals with diagnosed T2DM compared to those with higher insulin sensitivity and better capacity for defending glucose homeostasis.

Five animal studies investigating the effects of whole RR supplementation as treatment for the consequences of a HFD and/or obesity, T2DM, or MetSy measured markers of inflammation in various tissues (Table 2). In Zucker fa/fa rats prone to MetSy, and WT mice, RR decreased proinflammatory cytokines in serum increased by a HFD, while only a trend in decreased serum cytokines was observed in mice prone to obesity and T2DM fed a standard chow diet [32,36,38]. Regarding inflammation in organs, RR significantly attenuated proinflammatory cytokines in skeletal muscle, decreased macrophage infiltration in IngWAT, and decreased gene expression of inflammation and cytokines in the livers of WT mice fed a standard chow diet supplemented with RR [33,36,37]. Thus, whole RR supplementation has been demonstrated to decrease systemic inflammation as well as inflammation in skeletal muscle and the liver exacerbated by a HFD, while additionally has the capacity to decrease hepatic inflammation in the absence of excessive energy intake. 

An in vivo study investigated the effects of RR functional component (ellagic acid in RSF) supplementation to a HFSD on inflammation (Table 3) found that a HFSD, compared to a HFD regime, increased macrophage infiltration and pro-inflammatory gene expression in epididymal WAT (EpiWAT), which were both significantly attenuated by RSF supplementation. Additionally, the HFSD increased liver markers of inflammation and ER stress, which were also ameliorated by RSF supplementation [42]. The findings of this study provide additional evidence that RR may be beneficial in moderating the inflammatory effects of consuming a “Western-style” high-energy diet. 

### 3.6. Effects of RR Supplementation on Oxidative Stress and Cardiovascular Health

An overabundance of reactive oxygen species (ROS) can create an environment burdened by oxidative stress and inflammation and, when paired with chronic hyperglycemia and IR, can lead to glycation phenomena and damage of cardiovascular tissues and eventually to atherosclerosis [8]. All five animal studies utilizing RR functional components or extracts as a treatment for the consequences of high-energy diets measured markers of oxidative stress (Table 3). In three studies malondialdehyde (MDA), a marker of lipid peroxidation, was elevated by the HFFD and HFD diets [40,41,45]. Both RK and RKGC were effective at decreasing MDA in WAT, while serum MDA was decreased by all doses of RRE and liver MDA was decreased by the medium and high doses of RRE, and finally serum MDA was decreased by both doses of RK [40,41,45]. Interestingly, the Jia et al. (2011) study documented decreased serum MDA in spontaneously hypertensive rats (SHR) compared with WT rats, which was decreased further by both doses of ethyl acetate extract of red raspberries (EER) [49]. Endogenous antioxidant levels were altered by the high-energy diets in all studies included herein and improved with all RR treatments. Reduced glutathione (GSH) levels were elevated in WAT with RKGC supplementation, while expression of genes involved in antioxidant pathways was improved [40]. Tu et al. observed increased levels of serum and hepatic catalase (CAT), hepatic superoxide dismutase (SOD), and hepatic glutathione peroxidase (GPx) with all doses of RK, and increased serum SOD and GPx with the medium and high doses of RK [45]. Mehanna et al. found increased levels of serum GSH with both doses of RK and Kang et al. (2016) reported decreased levels of hepatic H_2_O_2_ with RSF supplementation [41,42]. In the SHR model, the inherent SOD deficiency was completely ameliorated by both doses of EER and even increased above levels of the WT rats. Additionally, EER supplementation led to improvements of blood pressure, serum nitric oxide (NO), and serum endothelin (ET) levels in the SHR [49]. Together, these studies provide evidence of the effectiveness of supplementation with functional RR components and extracts to improve oxidative stress and cardiovascular health. 

Of the animal studies investigating the effects of whole RR supplementation as a treatment for the consequences of a HFD and/or obesity, T2DM, or MetSy, two studies measured markers of oxidative stress and cardiac health (Table 2). Noratto et al. (2017) observed that mice prone to obesity and T2DM fed a standard chow diet supplemented with RR had reduced levels of ROS and increased endogenous antioxidant GPx in erythrocytes and liver tissue when compared to a standard diet alone [38]. Regarding cardiac health, RR supplementation decreased heart rate and improved significantly left ventricular hypertrophy (LVH) and interventricular septal wall thickening seen in Zucker fa/fa rats fed a HFD [32]. Thus, RR supplementation has the potential to improve the cardiovascular health in conditions of obesity, T2DM, and MetSy. While various bioactive compounds in RR, as well as other berries, extend anti-inflammatory properties, [50,51] it is unclear to what extent these compounds can reduce risk for T2DM.

Human trials to date have not provided significant evidence that long-term supplementation with RR has benefits for oxidative stress or cardiac health (Table 1). Two studies reported non-significant trends in decreased systolic blood pressure (SBP) from 250 g RR and 300 g SRC, respectively, with no effect on diastolic blood pressure (DBP) [30,46]. Serum levels of 8-isoprostane had a decreasing trend over-time in both experimental groups, those consuming SRC daily and those with restricted berry consumption [46]. However, there is evidence to support that intake of anthocyanins and flavones are associated with biomarkers that indicate reduced insulin resistance and inflammation in women [52]. Furthermore, berries were shown to reduce post-prandial insulin responses following wheat/rye bread consumption in healthy women, thus indicating the potential of berries in glucose regulation optimization through normalization of insulin secretion [53]. Moreover, evidence from pregnant women diagnosed with gestational diabetes demonstrated that consumption of raspberry leaf extract significantly reduced the amount of insulin administration needed to achieve glucose control [54]. From a prevention standpoint, there is evidence to suggest that increase in dietary polyphenol consumption can be protective against risk for T2DM in humans [55].

Conclusively, more research should be conducted in individuals with T2DM and MetSy to determine if there are benefits to cardiovascular health from consuming RR daily and delineate possible mechanisms of action. Interestingly, Kshatriya et al., demonstrated that phenolic-enriched raspberry fruit extract resulted in lower weight gain and elevated hepatic lipoprotein lipase in rodents thus offering some potential mechanistic insight as per potential modes of action towards obesity control and subsequent MetSy [56]. Additionally, in an effort to address the plausible question of extracts versus whole fruit, Koss-Mikołajczyk and colleagues, showed that food matrix embedded anthocyanins were more effective compared to isolated anthocyanins towards bioactivities such as antioxidant, cytotoxic, anti-genotoxic, thus lending indirect support to the food synergy concept, that suggests interactions among various phytochemicals as a decisive factor regarding the resulting bioactivity (qualitatively and quantitatively) of edible plants [57]. Moreover, in a more conservative in vivo model (T1DM), plant-derived polyphenol extract mixture was more effective towards ameliorating hyperglycemia, hyperlipidemia and histopathological decline in pancreas, kidney and liver compared to standard medication (insulin and metformin) [58]. Finally, in a recent systematic review of the evidence and meta-analysis, it is concluded that while polyphenol formulations for effectively supporting blood glucose levels control in T2DM patients exist, their further efficacy needs validation through human studies [59]. As underlined in the most recent version of the Dietary Guidelines for Americans, it is important for Nutritional Sciences research to address the gaps identified as per the mechanisms pertinent to the bioactivity of fruits, vegetables and grains and be thus more specific and precise in the future recommendations, more suited to the individual characteristics of the various populations [60].

## 4. Strengths, Limitations and Future Directions

Strengths of this review lie in choosing only peer reviewed RCTs that utilized an amount of RR feasible for daily consumption in human adults with T2DM, pre-diabetes, or MetSy; however, only three human studies were identified, and it appears that trials utilizing RR and/or their functional constituents as a treatment for T2DM or MetSy remain limited. Additionally, studies of longer duration and larger sample size are needed to increase statistical power, and measurement of HbA1c should be included to detect the long-term effects of hyperglycemia in individuals with T2DM. Strengths of the in vivo studies in this review include the utilization of whole RR or various functional components of RR as interventions for diet-induced metabolic dysfunction, and the utilization of all male animal models by most of the studies, controlling for confounding factors related to sex. One limitation of the selected studies is the high variation in diets, ranging from a standard chow diet in some studies to using various high-energy diets in others, some without a control diet. Additionally, although rodents have been shown to be appropriate models for studies related to human physiology, their size and energy requirements necessitate vastly different metabolic rates than humans, resulting in findings that may not be directly applicable to adult humans.

Future research trials are needed that utilize human participants with both pre-diabetes and T2DM to confirm the beneficial effects on metabolic health from both postprandial and long-term whole RR supplementation seen in the studies included in this review. This will allow for discernment of whether RR has the potential to prevent progression of pre-diabetes to T2DM, as well as investigate the efficacy of dietary lifestyle modifications for management of T2DM and IR prior to needing pharmaceutical intervention. These trials should focus on clarifying the dose-dependent relationships of RR supplementation while including measurements of plasma resistin levels and postprandial triglycerides to further explore the safety and efficacy of various doses and address the few concerning results from the studies herein. Additionally, human studies should explore the benefits of the dietary fibers in RR on the gut microflora of diabetics, with further examination of the bioavailability of polyphenols and their urolithin metabolites. Future in vivo studies should also focus on examining the dose-dependent relationships of RR supplementation to elucidate the optimal therapeutic dose while utilizing a standard chow diet as a control and a high-energy, “Western-style” diet that promotes metabolic dysfunction.

## 5. Conclusions

Various human and in vivo studies were examined in this literature review assessing effects of red raspberry (RR) supplementation on the metabolic dysregulation commonly seen in T2DM. Improvements in glucose handling and insulin sensitivity, adiposity, lipid profiles, ectopic lipid accumulation, inflammation, oxidative stress, and cardiac health were demonstrated at varying degrees by these studies.

According to available evidence from the studies included in this review and by translating RR doses into human-related intakes, it appears that including 1–1.5 cups of fresh or frozen red raspberries into a well-balanced, nutritious diet may provide beneficial effects on glucose, insulin, and lipid profiles, with a low risk of adverse effects, for individuals at high-risk for chronic disease or in need of mitigating T2DM progression. Collectively, the studies examined in this literature review provide evidence indicating that dietary red raspberries and their bioactive components may offer clinically beneficial effects for the prevention and management of chronic diseases, through improvements in glucose handling and insulin sensitivity, adiposity, lipid profiles, ectopic lipid accumulation, inflammation, oxidative stress, and cardiac health. However, more studies are needed to obtain conclusive results.

## Figures and Tables

**Table 1:** Summary of randomized controlled trials investigating the effects of dietary whole red raspberries in humans with metabolic dysfunction.

Reference (Year)	Participants	Study Design	Treatment	Duration	Significant Findings (*p* < 0.05)
Schell et al. (2019)	Adults with T2DM: Phase 1 (*n* = 25) Phase 2 (*n* = 22)	RCT, crossover	Frozen RR: Phase 1—250 g RR (RR-250) or 85 g banana (control) with a HF challenge breakfast meal Phase 2—250 g RR (RR-250) per day or control as afternoon snack	Phase 1 (acute): 4 h (×2 d plus 1-wk washout) Phase 2 (long-term): 4 wks (×2 plus 2-wk washout)	Phase 1 (RR-250 vs. control): ↓ SG and ↓ SG AUC at 2- and 4 h post-meal; ↓ serum TG at 4 h post-meal (*p* < 0.1); ↓ IL-6 and ↓ hs-TNF-α at 4 h post-meal Phase 2 (RR-250 vs. control): ↓ IL-6; ↓ hs-TNF-α; ↓ SBP (*p* < 0.1)
Xiao et al. (2019)	Adults with Pre-DM and overweight or obesity with IR (*n* = 21) and Healthy adults (reference group, *n* = 11)	RCT, crossover	Frozen RR: 0 g RR (control), 125 g RR (RR-125), or 250 g RR (RR-250) with a HF challenge breakfast meal	Acute: 24 h (×3 d and at least 3 d apart)	*Pre-DM Group* RR-125 vs. control: ↓ PG 30 min peak post-meal; ↓ PIns 2 h AUC; Peak PIns shifted to 1 h vs. 30 min RR-250 vs. control: ↓ PG 30 min peak and at 1 h post-meal, ↓ PG and PIns 2 h AUC, ↓ PIns over 24-h, ↓ PIns at 30 min post-meal; Peak PIns shifted to 1 h vs. 30 min RR-250 vs. RR-125 and control: ↑ plasma TG at 2–6 h post-meal *Reference Group* RR-125 and 250 vs. control: ↑ PG at 2 h post-meal (averting dip below baseline seen in control)
Puupponen-Pimiä et al. (2013)	Adults with MetSy (*n* = 32)	RCT	SRC Group (*n* = 20): 300 g SRC per d (100 g fresh strawberry puree + 100 g frozen RR + 100 g frozen cloudberries) Control Group (*n* = 12): Restricted berry consumption	Lead-In (SRC and control): 4 wk restricted berry consumption (maximum: 1 dL/d) Intervention: 8 wks Recovery: 4 wk restricted berry consumption	SRC vs. control: ↑ leptin over time SRC: 15 participants produced urolithins (5 non-producers) with concurrent alteration of gut microflora Control: 0 participants produced urolithins

RCT, randomized controlled trial; RR, red raspberries; HF, high-fat; IR, insulin resistance; PG, plasma glucose; SG, serum glucose; PIns, plasma insulin; AUC, area under the curve; SBP, systolic blood pressure; TG, triglycerides; Pre-DM, pre-diabetes; T2DM, type 2 diabetes mellitus; MetSy, metabolic syndrome; IL-6, interleukin-6; hs-TNF-α, high sensitivity tumor necrosis factor alpha; SRC, strawberries, raspberries and cloudberries; Tx, treatment; min, minutes; h, hour; d, day; wk, week; wks, weeks; y, year; ↓, decrease; ↑, increase; ↔, no effect. Underline: for distinction purposes. Italics: for concept distinction.

**Table 2:** Summary of animal studies investigating the effects of dietary whole red raspberries on metabolic conditions.

Reference (Year)	Animal Model (Health Condition; Sex)	Dietary Treatments	Duration	Significant Findings (*p* < 0.05)
Kirakosyan et al. (2018)	Zucker Fatty rats (obesity & MetSy; male)	10% below *ad libitum* intake HFD (*n* = 12): Base high-fat diet (17% kcal Pro, 45% kcal CHO, 38% kcal fat) HFD-RR (*n* = 12): Base diet + 2% freeze-dried RR powder [Hypercaloric]	12 wks	HFD-RR vs. HFD: ↓ FSG; ↓ serum TC and TG; ↑ cardiac gene expression of *Adipor1* and *ApoE*; ↓ cardiac gene expression *LPL*; ↓ plasma IL-6, TNF-α, and 8-isoprostane; ↓ cardiac gene expression of various key genes involved in lipid and glucose dynamics, insulin signaling, T2DM, inflammation, immunity, and apoptosis; ↓ LVH and IVSth at 8 wks; ↓ HR
Zhao et al. (2018)	AMPKα1 knockout mice (AMPKα1^−/−^; male) AMPKα1^fl/fl^ mice (WT; male)	*Ad libitum* WT (*n* = 6): High-fat diet (60% kcal fat) WT-RR (*n* = 6): HFD + 5% freeze-dried RR powder per dry feed weight AKO (*n* = 6): HFD AKO-RR (*n* = 6): HFD + 5% freeze-dried RR powder per dry feed weight [Hypercaloric]	10 wks	WT-RR vs. WT: ↓ excess body weight gain WT-RR vs. WT (in *Gas*): ↑ p-AMPKα and ↑ p-/t-AMPKα; ↑ Glut4 protein and Glut4 gene expression; ↓ p-PKCθ; ↑ p-AKT and p-/t-AKT; ↓ TG; ↓ *TNF-α*, *IL-1β*, *IL-6*, and *IL-18* gene expression; ↓ p-p65 and p-/t-p65; ↓ p-JNK; ↑ CytC; ↑ *PGC1*α, *NRF1*, and *CPT1* gene expression WT-RR vs. WT (in *TibA*): ↓ fat area (↔ in AKO or AKO-RR for any parameter)
Zou et al. (2018)	AMPKα1 knockout C57BL/6 mice (AMPKα1^−/−^; male) C57BL/6 AMPKα1^flox/flox^ mice (WT; male)	*Ad libitum* WT (*n* = 6): High-fat diet (60% kcal fat) WT-RR (*n* = 6): HFD + 5% freeze-dried RR powder per dry feed weight AKO (*n* = 6): HFD AKO-RR (*n* = 6): HFD + 5% freeze-dried RR powder per dry feed weight [Hypercaloric]	10 wks	WT-RR vs. WT/AKO/AKO-RR: ↓ body weight; ↑ BAT mass; ↓ IngWAT and EpiWAT mass; ↓ glucose AUC; ↓ SIns; ↓ serum TG; ↓ serum FFAs; ↓ serum TC; ↑ VO_2_; ↓ RER; ↑ heat production; ↑ O_2_ consumption in IngWAT; ↑ thermogenic gene expression in BAT (*UCP1*, *PRDM16*, *PGC1α*, *Cidea*, and *Cox7a1*); ↑ thermogenic proteins in BAT (UCP1, PRDM16, and CtyC); ↑ p-AMPKα and p-/t-AMPKα in BAT; ↓ diameter and number of adipocytes in IngWAT and EpiWAT; ↑ beige adipocytes and UCP1-positive areas in IngWAT; ↑ thermogenic gene expression in IngWAT (*UCP1*, *PRDM16*, *PGC1α*, *Elovl3*, and *Cox7a1*); ↑ gene expression of beige adipocyte markers in IngWAT (*CD137*, *Tbx1*, and *Tmem26*); ↑ thermogenic proteins in IngWAT (UCP1 and PRDM16); ↑ p-AMPKα, t-AMPKa, and p-/t-AMPKα in IngWAT WT-RR vs. WT: ↑ VCO_2_ (*p* < 0.1); ↑ thermogenic protein CytC in IngWAT (↔ in AKO or AKO-RR for any parameter)
Xing et al. (2018)	C57BL/6J mice (WT; male)	*Ad libitum* Control (*n* = 8): Standard chow (10% kcal fat) RR (*n* = 8): Standard chow + 5% freeze-dried RR powder per dry feed weight HFD (*n* = 8): High-fat diet (60% kcal fat) HFD-RR (*n* = 8): HFD + 5% freeze-dried RR powder per dry feed weight [Hypercaloric]	12 wks	HFD vs. control/RR/HFD-RR: ↑ IngWAT and EpiWAT/body weight; ↑ diameter and ↓ number of adipocytes; ↑ macrophages; ↑ gene expression of *MCP1*, *CD14*, and *CD68*; ↑ p-p65; ↓ IĸBα; ↑ IL-1β, IL-6, and IL-18; ↓ p-AKT and p-/t-AKT; ↓ Glut4; ↑ p-IRS1 and p-/t-IRS1; ↑ p-PKCθ and p-/t-PKCθ; ↓ PGC1α, FNDC5, p-p38, p-/t-p38, p-ERK, and p-/t-pERK; ↓ thermogenic gene expression (*UCP1*, *CytC*, *Cidea*, and *Elovl3*); ↓ thermogenic proteins (UCP1, UCP2, and CytC); ↓ p-AMPK and p-/t-AMPK; ↓ Sirt1 HFD-RR vs. HFD: ↓ IngWAT/body weight; diameter and ↑ number of adipocytes; ↓ macrophages; ↓ gene expression of *MCP1*, *CD14*, and *CD68*; ↑ IĸBα; ↓ IL-1β, IL-6, and IL-18; ↑ p-AKT and p-/t-AKT; ↑ Glut4; ↓ p-IRS1 and p-/t-IRS1; ↓ p-PKCθ and p-/t-PKCθ; ↑ PGC1α, FNDC5, p-p38, p-/t-p38, p-ERK, and p-/t-pERK; ↑ thermogenic gene expression (*UCP1*, *Cidea*, and *Elovl3*); ↑ thermogenic proteins (UCP1 and CytC); ↑ p-AMPK and p-/t-AMPK; ↑ Sirt1 HFD vs. control: ↑ p-/t-p65
Zhu et al. (2018)	C57BL/6 mice (WT; male)	*Ad libitum* Control (*n* = 10): Standard chow (10% kcal fat) RR (*n* = 10): Standard chow + 5% freeze-dried RR powder per dry feed weight HFD (*n* = 10): High-fat diet (60% kcal fat) HFD-RR (*n* = 10): HFD + 5% freeze-dried RR powder per dry feed weight [Hypercaloric]	12 wks	RR vs. control: ↑ p-IRS-1 & p-/t-IRS-1; ↑ p-AKT (*p* < 0.1); ↑ p-/t-AKT; ↓ hepatic IL-1β & IL-18 HFD/HFD-RR vs. control/RR: ↑ BMI; ↑ adiposity; ↓ RER; ↑ t-AKT; ↑ liver TG HFD vs. control/RR: ↓ VO2; ↓ VCO2; ↑ FSG; ↑ glucose AUC; ↓ insulin sensitivity; ↑ hepatic lipid droplets; ↑ serum TG; ↑ hepatic CASP1; ↑ hepatic IL-1β & IL-18; ↑ hepatic gene expression of *NLRP3*, *CASP1*, *IL-1β*, *IL-18*, *PGC1a*, *NRF1*, *CytB*, *Cox4i1*, & *Cs* HFD-RR vs. HFD: ↓ excess body weight gain; ↑ VO2 (*p* < 0.1); ↑ VCO2; ↓ FSG; ↓ glucose AUC; ↑ insulin sensitivity; ↑ p-IRS-1 & p-/t-IRS-1; ↑ p-/t-AKT; ↓ hepatic lipid droplets; ↓ serum TG (*p* < 0.1); ↓ hepatic CASP1; ↓ hepatic IL-1β & IL-18; ↓ hepatic gene expression of *NLRP3*, *CASP1*, *IL-1β*, *IL-18*, *PGC1α*, *NRF1*, *CytB*, *Cox4i1*, & *Cs*
Luo et al. (2017)	C57BL/6J mice (WT; male)	*Ad libitum* LFD (*n* = 8): Low-fat diet (10% kcal fat, 70% kcal CHO) HFD (*n* = 8): High-fat diet (45% kcal fat, 20% kcal sucrose, 1% cholesterol) HFD-RPC (*n* = 8): HFD + RPC (2.5% kcal) HFD-RJC (*n* = 8): HFD + RJC (2.5% kcal) [Hypercaloric]	10 wks	HFD/HFD-RPC/HFD-RJC vs. LFD: ↑ energy intake; ↑ liver weight; ↑ IngWAT weight HFD vs. LFD: ↑ body weight; ↑ liver lipid percentage; ↑ FSG; ↑ Sins; ↔ hepatic gene expression of *Hmox1* or *Lipe* HFD-RPC/HFD-RJC vs. HFD: ↓ body weight; ↓ liver lipid percentage HFD-RJC vs. HFD: ↓ SIns HFD-RJC vs. LFD/HFD/HFD-RPC: ↑ hepatic gene expression of *Hmox1* & *Lipe*
Noratto et al. (2017)	Db/Db mice (obesity & T2DM; male and female)	*Ad libitum* Control (*n* = 15): Standard chow (20.5% kcal Pro, 63.6% kcal CHO, 15.9 % kcal fat) RR (*n* = 15): Standard chow + freeze-dried RR (20.5% kcal Pro, 53.7% kcal CHO, 15.9% kcal fat, 9.8% kcal RR) [Isocaloric]	8 wks	RR vs. control: ↑ plasma TC & LDL-c with ↔ in atherogenic index; ↑ plasma resistin; ↓ ROS, ↑ GPx, & ↑ GPx/SOD ratio in blood erythrocytes; ↑ GPx & ↑ GPx/SOD ratio activity in liver

RR, red raspberries; HFD, high-fat diet; LFD, low-fat diet; Pro, protein; CHO, carbohydrate; RPC, raspberry puree concentrate; RJC, raspberry juice concentrate; AMPKα1, AMP-activated protein kinase alpha 1; AKO, AMPKα1 knockout, WT, wild type; Gas, Gastrocnemius muscle; TibA, Tibialis anterior muscle; WAT, white adipose tissue; BAT, brown adipose tissue; IngWAT, inguinal WAT; EpiWAT, epididymal WAT; FSG, fasting serum glucose; SIns, serum insulin; AUC, area under the curve; TG, triglycerides; FFAs, free fatty acids; TC, total cholesterol; LDL-c, low-density lipoprotein cholesterol; ApoE, apolipoprotein E; T2DM, type 2 diabetes mellitus; MetSy, metabolic syndrome; LVH, left ventricular hypertrophy; IVSth, interventricular septal thickness; HR, heart rate; IL-6, interleukin-6; IL-1β, interleukin-1 beta; IL-18, interleukin-18; TNF-α, tumor necrosis factor alpha; JNK, c-Jun N-terminal kinase; Adipor1, adiponectin receptor 1; LPL, lipoprotein lipase; Glut4, glucose transporter 4; PKCθ, protein kinase C theta; AKT, protein kinase B; IRS-1, insulin receptor substrate 1; CytC, cytochrome C; PGC1α, peroxisome proliferator-activated receptor gamma coactivator 1 alpha; NRF1, nuclear respiratory factor 1; CPT1, carnitine palmitoyltransferase 1; NLRP3, NLR family pyrin domain containing 3; CASP1, caspase 1; UCP1, uncoupling protein 1; UCP2, uncoupling protein 2; PRMD16, PR domain containing 16; Cidea, cell death-inducing DFFA-like effector A; CytB, cytochrome B; Cox4i1, cytochrome C oxidase subunit IV; Cox7a1, cytochrome C oxidase subunit VIIa polypeptide 1; Elovl3, very long-chain fatty acids protein 3; Cs, citrate synthase; CD137, cluster of differentiation 137; Tbx1, T-box 1; Tmem26, transmembrane protein 26; Lipe, hormone sensitive lipase; Hmox1, heme oxygenase 1; MCP1, monocyte chemoattractant protein 1; CD14, cluster of differentiation 14; CD68, cluster of differentiation 68; IĸBα, inhibitor ĸB alpha; ERK, extracellular regulated protein kinase; p65, nuclear factor NF-kappa-B p65 subunit; p38, mitogen-activated protein kinase; Sirt1, sirtuin 1; ROS, reactive oxygen species; GPx, glutathione peroxidase; SOD, superoxide dismutase; VO_2_, volume of oxygen consumption per minute; VCO_2_, volume of carbon dioxide production; RER, respiratory exchange ratio; p-, phosphorylated; t-, total; min, minutes; h, hour; d, day; wk, week; y, year; ↓, decrease; ↑, increase; ↔, no effect. Underline: for distinction purposes. Italics: for concept distinction.

**Table 3:** Summary of animal studies investigating the effects of extracts or functional constituents of red raspberries on metabolic conditions.

Reference (Year)	Animal Model (Health Condition; Sex)	Dietary Treatments	RR Constituent	Duration	Significant Findings (*p* < 0.05)
Attia et al. (2019)	Wistar rats (WT, male)	LFD (*n* = 10): Standard chow (26% kcal Pro, 60% kcal CHO, 8% kcal fibers, 5% kcal fat) HFFD (*n* = 10): High-fat, high- fructose diet (21% kcal Pro, 60% kcal CHO, 3% kcal fibers, 15% kcal fat HFFD-RK * (*n* = 10): HFFD + 55 mg/kg RK HFFD-GC *^#^ (*n* = 10): HFFD + 600 mg/kg GC HFFD-RKGC * (*n* = 10): HFFD + 55 mg/kg RK +600 mg/kg GC powders [Hypercaloric] * RK and GC Tx were administered via oral gavage ^#^ Significant results not discussed in current review	Raspberry ketone (RK)	8 wks	HFFD/HFFD-RK/HFFD-RKGC vs. LFD: ↑ body weight; ↑ fasting SIns; ↑ HOMA-IR; ↓ gene expression of *NRF2* in EpiWAT; ↓ p-AKT in VAT and EpiWAT; ↓ p-IRS1 in EpiWAT; ↑ number of adipocytes HFFD/HFFD-RK vs. LFD: ↑ FSG; ↑ serum TG, TC, LDL-c, and FFAs; ↓ serum HDL-c; ↑ SREBP1c gene expression in VAT and EpiWAT; ↓ gene expression of *NRF2* in VAT; ↓ GSH in VAT; ↓ p-IRS1 in VAT; ↓ Glut4 in EpiWAT; ↑ serum STAT3 in VAT and EpiWAT HFFD vs. LFD: ↑ MDA in VAT and EpiWAT; ↓ Glut4 in VAT; ↑ serum leptin HFFD-RK/HFFD-RKGC vs. HFFD: ↓ body weight; ↓ FSG; ↓ fasting SIns; ↓ HOMA-IR; ↓ serum TG, TC, LDL-c, and FFAs; ↑ serum HDL-c; ↓ *SREBP1c* gene expression in VAT and EpiWAT; ↑ gene expression of *NRF2* in VAT and EpiWAT; ↓ MDA in VAT and EpiWAT; ↑ p-AKT in VAT and EpiWAT; ↑ p-IRS1 in VAT and EpiWAT; ↑ Glut4 in VAT; ↓ serum STAT3 in VAT and EpiWAT; ↓ number of adipocytes HFFD-RKGC vs. HFFD: ↓ serum leptin HFFD-RKGC vs. HFFD/HFFD-RK: ↑ GSH in VAT; ↑ Glut4 in EpiWAT HFFD-RKGC vs. LFD/HFFD/HFFD-RK: ↑ GSH in EpiWAT HFFD-RK vs. LFD/HFFD/HFFD-RKGC: ↓ serum leptin; ↓ adipocyte area
Tu et al. (2019)	C57BL/6J mice (WT, male)	*Ad libitum* Control (*n* = 8): Standard chow (315 kcal/100 g feed) HFD (*n* = 8): High-fat diet (409 kcal/100 g feed) HFD-RL * (*n* = 8): HFD +100 mg/kg freeze-dried RRE HFD-RM * (*n* = 8): HFD +200 mg/kg freeze-dried RRE HFD-RH * (*n* = 8): HFD +300 mg/kg freeze-dried RRE [Hypercaloric] * RRE Tx administered via gastric gavage.	Red raspberry extract (RRE)	8 wks	HFD vs. control: ↑ total body weight; ↑ body weight gain; ↓ serum CAT; ↓ serum & hepatic SOD; ↑ serum and hepatic MDA; ↓ hepatic GPx; ↑ serum and hepatic TC & TG; ↑ hepatic total bile acids HFD/HFD-RL/HFD-RM/HFD-RH vs. control: ↑ body weight gain; ↑ EpiWAT and PeriAT weight; ↑ EpiWAT and PeriAT fat index HFD/HFD-RL vs. control: ↑ EpiWAT cell size; ↑ hepatic MDA; ↑ serum AST HFD-RL/HFD-RM/HFD-RH vs. HFD/control: ↑ serum and hepatic CAT; ↑ hepatic SOD HFD-RL/HFD-RM/HFD-RH vs. HFD: ↓ body weight gain; ↓ EpiWAT and PeriAT weight; ↓ serum MDA; ↑ hepatic GPx; ↓ hepatic TC and TG; ↑ hepatic gene expression of *Pparα* and *Pnpla2*; ↓ hepatic gene expression of *Mups*, *Hmox1*, *Gapdh*, *HMGCR*, *Ldlr*, and *Acsl3* HFD-RM/HFD-RH vs. HFD/control: ↑ serum GPx HFD-RM/HFD-RH vs. HFD: ↓ EpiWAT cell size; ↑ serum SOD; ↓ hepatic MDA; ↓ serum TC& TG HFD-RM vs. HFD: ↓ PeriAT fat index HFD-RH vs. HFD: ↓ total body weight; ↓ EpiWAT fat index; ↓ hepatic total bile acids
Mehanna et al. (2018)	Wister albino rats (WT, male)	Control (*n* = 8): Standard chow HFD (*n* = 8): High-fat diet (87.7% kcal standard chow, 10% kcal pork fat, 2% kcal cholesterol, 3% kcal bile salts) HFD-RKL* (*n* = 8): HFD + 250 mg/kg RK HFD-RKH * (*n*=8): HFD + 500 mg/kg RK [Isocaloric] * RK Tx administered via gastric gavage from wk 9 to wk 12 (4 wks).	Raspberry ketone (RK)	12 wks (RK Tx for 4 wks)	HFD vs. control: ↑ liver index; ↑ serum TC; ↑ serum LDL-c; ↓ serum HDL-c; ↑ serum leptin; ↑ WAT gene expression of *leptin*; ↓ WAT gene expression of *AQP-7* HFD/HFD-RKH vs. control: ↑ serum apelin & visfatin; ↓ serum adiponectin; ↑ WAT gene expression of *apelin*, *apelin receptor*, & *visfatin*; ↓ WAT gene expression of *adiponectin*; ↑ adipocyte diameter HFD/HFD-RKL/HFD-RKH vs. control: ↑ body weight gain; ↑ adipose tissue index; ↑ serum ALT; ↑ serum AST; ↑ FSG; ↑ fasting Sins; ↑ HOMA-IR, ↓ QUICKI; ↑ serum TG; ↓ serum GSH; ↑ serum MDA; ↑ hepatic steatosis HFD-RKL/HFD-RKH vs. HFD: ↓ body weight gain; ↓ adipose tissue index; ↓ liver index; ↓ serum ALT; ↓ serum AST; ↓ FSG; ↓ fasting Sins; ↓ HOMA-IR, ↑ QUICKI; ↓ serum TG, TC, & LDL-c; ↑ serum HDL-c; ↑ serum GSH; ↓ serum MDA; ↓ serum leptin; ↓ WAT gene expression of *leptin*; ↑ WAT gene expression of *AQP-7*; ↓ adipocyte diameter HFD-RKL vs. HFD: ↓ hepatic steatosis HFD-RKL vs. HFD/HFD-RKH: ↓ serum apelin & visfatin; ↑ serum adiponectin; ↓ WAT gene expression of *apelin*, *apelin receptor*, & *visfatin*; ↑ WAT gene expression of *adiponectin* HFD-RKL vs. HFD-RKH: ↓ serum ALT; ↓ serum AST; ↓ FSG; ↓ fasting Sins; ↓ HOMA-IR, ↑ QUICKI
Kang et al. (2016)	C57BL/6 mice (WT, male)	*Ad libitum* HFD (*n* = 6): High-fat diet (41% kcal fat) HFSD (*n* = 6): High-fat, high-sucrose diet (37% kcal sucrose) HFSD-R (*n* = 6): HFSD + 3% RSF (equivalent to 0.03% ellagic acid) [Isocaloric]	Raspberry seed flour (RSF)	12 wks	HFSD vs. HFD: ↑ body weight; ↑ liver weight; ↑ EpiWAT weight; ↑ VAT weight; ↑ plasma TC & LDL-c; ↓ plasma HDL-c; ↑ fasting PIns; ↑ HOMA-IR; ↑ glucose & insulin AUCs; ↑ hepatic gene expression of *SCD-1*, *LPL*, *DGAT2*, & *G6Pase*; ↑ hepatic H_2_O_2_ (*p* < 0.01); ↑ macrophage infiltration in EpiWAT; ↑ adipocyte diameter in EpiWAT (*p* < 0.01) HFSD/HFSD-R vs. HFD: ↑ liver TG; ↑ hepatic gene expression of *ChREBP*, *SREBP1c*, & *PEPCK* HFSD-R vs. HFSD: ↓ plasma TG (*p* < 0.1), TC, & LDL-c; ↑ plasma HDL-c; ↓ FPG & fasting PIns (*p* < 0.01); ↓ HOMA-IR; ↓ insulin AUC (*p* < 0.01); ↓ hepatic gene expression of *SCD-1*, *LPL*, *DGAT2*, & *G6Pase*; ↓ hepatic p-JNK, p-p38, & p-eIF2α; ↓ hepatic H_2_O_2_ (*p* < 0.01); ↓ macrophage infiltration in EpiWAT; ↓ adipocyte diameter in EpiWAT (*p* < 0.01); ↑ plasma adiponectin (*p* < 0.01); ↑ EpiWAT gene expression of *adiponectin*; ↓ EpiWAT gene expression of *IL-6* (*p* < 0.001) & *IL-8*, *F4/80*, *TNF-α*, & *MCP1* (*p* < 0.01)
Jia et al. (2011)	SHR rats (Hypertension, male) WKY rats (WT, male)	*Ad libitum* Control * (*n* = 8): Standard chow SHR ** (*n* = 8): Standard chow EERL **^#^ (*n* = 8): Standard chow + 100 mg/kg/d EER EERH **^#^ (*n* = 8): Standard chow + 200 mg/kg/d EER [Isocaloric] * WKY rats ** SHR rats ^#^ EER Tx administered via gastric gavage	Ethyl acetate extract of RR (EER)	5 wks	SHR: ↑ SBP at wk 4; ↑ SBP at wk 5 (*p* < 0.01) SHR vs. control: ↑ serum ET, ↓ serum SOD (*p* < 0.01); ↓ serum MDA (*p* < 0.01) EERL: ↓ SBP at wks 2-3; ↓ SBP at wks 4-5 (*p* < 0.01) EERL vs. control: ↑ serum NO; ↑ serum SOD (*p* < 0.01); ↓ serum MDA (*p* < 0.01) EERL vs. SHR: ↓ SBP at wks 2-5 (*p* < 0.01); ↑ serum NO (*p* < 0.01); ↑ serum SOD (*p* < 0.01) EERH: ↓ SBP at wks 2-5 (*p* < 0.01) EERH vs. control: ↑ serum SOD (*p* < 0.01); ↓ serum MDA (*p* < 0.01) EERH vs. SHR: ↓ SBP at wks 2-5 (*p* < 0.01); ↓ serum ET; ↑ serum SOD (*p* < 0.01); ↓ serum MDA

RK, raspberry ketone; RSF, raspberry seed flour; SHR, spontaneously hypertensive rats; WKY, Wistar Kyoto rats; EER, ethyl acetate extract of RR; RRE, red raspberry extract; HFD, high-fat diet; HFFD, high-fat, high-fructose diet; HFSD, high-fat, high-sucrose diet; LFD, low-fat diet; Pro, protein; CHO, carbohydrate; GC, *Garcinia Cambogia*; WT, wild type; WAT, white adipose tissue; VAT, visceral adipose tissue; EpiWAT, epididymal WAT; PeriAT, perirenal adipose tissue; IR, insulin resistance; FPG, fasting plasma glucose; FSG, fasting serum glucose; PIns, plasma insulin; SIns, serum insulin; AUC, area under the curve; QUICKI, quantitative insulin sensitivity check index; SBP, systolic blood pressure; NO, nitric oxide; ET, endothelin; TG, triglycerides; TC, total cholesterol; LDL-c, low-density lipoprotein cholesterol; HDL-c, high-density lipoprotein cholesterol; SREBP1c, sterol regulatory element-binding protein 1; ChREBP, carbohydrate response element-binding protein; STAT3, signal transducer and activator of transcription 3; ALT, alanine aminotransferase; AST, aspartate aminotransferase; IL-6, interleukin-6; IL-8, interleukin-8; IL-1β, interleukin-1 beta; IL-18, interleukin-18; TNF-α, tumor necrosis factor alpha; JNK, c-Jun N-terminal kinase; LPL, lipoprotein lipase; Glut4, glucose transporter 4; G6Pase, glucose 6-phosphatase; PEPCK, phosphoenolpyruvate carboxyl kinase; PKCθ, protein kinase C theta; AKT, protein kinase B; IRS-1, insulin receptor substrate 1; CytC, cytochrome C; SCD-1, stearoyl CoA desaturase-1; DGAT2, diacylglycerol acyltransferase 2, AQP-7, aquaporin-7; NRF1, nuclear respiratory factor 1; Cidea, cell death-inducing DFFA-like effector A; Hmox1, heme oxygenase 1; Pparα, peroxisome proliferator-activated receptor alpha; Mups, major urinary proteins; Gapdh, glyceraldehyde-3-phosphate dehydrogenase gene; HMGCR, hydroxymethylglutaryl coenzyme A reductase; Ldlr, low-density lipoprotein receptor gene; Pnpla2, adipose triglyceride lipase gene; Acsl3, long-chain acyl-CoA synthetase gene; MCP1, monocyte chemoattractant protein 1; F4/80, a marker of monocytes; p38, mitogen-activated protein kinase; eIF2α, eukaryotic translation initiation factor 2A; MDA, malondialdehyde; GPx, glutathione peroxidase; GSH, glutathione reduced form; CAT, catalase; SOD, superoxide dismutase; HOMA-IR, homeostatic assessment of insulin resistance; Tx, treatment; p-, phosphorylated; t-, total; min, minutes; h, hour; d, day; wk, week; y, year; ↓, decrease; ↑, increase; ↔, no effect. Underline: for distinction purposes. Italics: for concept distinction.

## Data Availability

The data on studies presented in this study are available on request from the corresponding author.
